# Emergence of *mcr-3 carrying Escherichia coli* in Diseased Pigs in South Korea

**DOI:** 10.3390/microorganisms8101538

**Published:** 2020-10-06

**Authors:** Abraham Fikru Mechesso, Dong Chan Moon, Hee Young Kang, Hyun-Ju Song, Su-Jeong Kim, Ji-Hyun Choi, Mi Hyun Kim, Seok Hyeon Na, Ha-Young Kim, Byeong Yeal Jung, Soon-Seek Yoon, Suk-Kyung Lim

**Affiliations:** 1 Bacterial Disease Division, Animal and Plant Quarantine Agency, 177 Hyeksin 8-ro, Gimcheon-si, Gyeongsangbuk-do 39660, Korea; abrahamf@korea.kr (A.F.M.); ansehdcks@korea.kr (D.C.M.); kanghy7734@korea.kr (H.Y.K.); shj0211@korea.kr (H.-J.S.); kimsujeong27@gmail.com (S.-J.K.); wlgus01@korea.kr (J.-H.C.); kimmh940301@naver.com (M.H.K.); nash8090@korea.kr (S.H.N.); kimhy@korea.kr (H.-Y.K.); jungby@korea.kr (B.Y.J.); yoonss24@korea.kr (S.-S.Y.); 2 Animal Disease Diagnostic Division, Animal and Plant Quarantine Agency, 177 Hyeksin 8-ro, Gimcheon-si, Gyeongsangbuk-do 39660, Korea

**Keywords:** colistin, *Escherichia coli*, *mcr*-*3* gene, plasmid, resistance

## Abstract

We examined the prevalence and molecular characteristics of *mcr-3* carrying colistin-resistant *Escherichia coli* among cattle, pig, and chicken isolates in South Korea. Among a total of 185 colistin-resistant *E. coli* isolates determined in this study (47 from cattle, 90 from pigs, and 48 from chicken), PCR amplification detected *mcr*-*3* genes in 17 isolates predominantly from diseased pigs. The *mcr*-*3* genes were characterized as *mcr-3.1* in 15 isolates and *mcr*-*3.5* in 2 isolates. The *mcr-3* gene was transferred to the *E. coli* J53 recipient strain from more than 50% of the *mcr*-*3*-carrying isolates. The *mcr*-*3.1* and *mcr*-*3.5* genes were identified predominantly in IncHI2 and IncP plasmids, respectively. Multi-locus sequence typing analysis revealed eight previously reported sequence types (ST), including ST1, ST10, and ST42. We identified isolates with similar pulsed-field gel electrophoresis patterns from diseased pigs in three farms. Besides, the isolates carried various virulence factors and demonstrated resistance to multiple antimicrobials, including β-lactams and quinolones. Further, the *mcr*-*3.5* encodes three amino acid substitutions compared with *mcr*-*3.1*. To the best of our knowledge, this is the first report of pathogenic *E. coli* carrying *mcr*-*3.5* in South Korea, which implies that *mcr*-*3* variants may have already been widely spread in the pig industry.

## 1. Introduction

Colistin is considered one of the last-resort antimicrobial agents against multi-drug resistant Gram-negative bacterial infections. The emergence of *mcr*-harboring colistin-resistant *Escherichia coli* presented a serious public health risk. Among the ten *mcr* genes identified so far i.e., *mcr*-*1* up to *mcr*-*10*, the *mcr*-*3* genes have been distributed worldwide [1]. Recent studies in South Korea (Korea), identified *mcr*-*3*-carrying *E. coli* isolates from food-producing animals [2,3]. However, both studies investigated limited numbers of isolates from specific provinces. Besides, although the *mcr-3* gene is subjected to constant evolution due to the impacts of unknown selective pressure in the environment, animals, and humans [4], no attempt has been made so far to determine the *mcr-3* variants in bacteria isolated in Korea. Consequently, we undertook this study to provide new knowledge on the prevalence and molecular characteristics of *mcr*-*3* variants in *E*. *coli* isolated from food-producing animals throughout Korea between 2005 and 2018.

## 2. Materials and Methods

### 2.1. Identification of Colistin-Resistant E. coli

*E. coli* isolates were recovered from healthy and diseased animals (i.e., cattle, chicken, and pigs), and their carcasses during a nationwide surveillance study on antimicrobial susceptibility conducted between 2005 and 2018. The minimum inhibitory concentration (MIC) of colistin was determined by the broth microdilution method [5] in KRNV5F Sensititre Panel following the manufacturer’s instruction (Trek Diagnostic Systems, Waltham, MA, USA). The MIC values were interpreted according to the EUCAST breakpoint (>2 µg/mL). PCR amplification was performed to investigate the *mcr-3* gene carriage of isolates exhibiting colistin resistance using primer pairs and PCR conditions described previously [6].

### 2.2. Conjugation Assay

Conjugation was performed using a filter mating method with azide-resistant *E. coli* J53 as the recipient strain [7]. The transconjugants were confirmed by PCR detection of the *mcr-3* genes and were investigated for their MICs as described above.

### 2.3. Molecular Characterization of mcr-3 carrying E. coli

A PCR-based replicon typing kit (Diatheva, Fano, Italy) and a multiplex PCR assay [8] were used to identify the plasmid replicon types and virulence factor genes, respectively. Pulsed-field gel electrophoresis of *mcr-3* positive isolates was conducted using genomic DNA prepared in agarose blocks, digested with Xbal enzyme (TaKaRa, Shiga, Japan), as described previously [9]. The banding profiles were analyzed using Bionumerics software and the genetic relatedness of the isolates was calculated using the unweighted pair-group method. Besides, molecular typing of *mcr-3* carrying isolates was carried out according to the protocols specified at the *E. coli* multilocus sequence typing website [10].

### 2.4. Whole-Genome Sequencing

Whole-genome sequencing was conducted to investigate the immediate genetic environment and amino acid sequences of *mcr-3* genes (PacBio RSII platform, Pacific Biosciences, Menlo Park, CA, USA). Complete sequences of the chromosomes and plasmids of strain V01-E02-025, V01-E02-51, and V01-R02-053 have been deposited into GenBank under the accession no. (CP049943, CP049944), (CP049299, CP049300), and (CP049086, CP049087), respectively.

## 3. Results and Discussion

A total of 14,631 *E. coli* isolates were obtained from healthy and diseased animals (i.e., cattle, chicken, and pigs), and their carcasses during 2005−2018 (Table 1). The overall prevalence of colistin-resistant *E. coli* was less than 5%. In our previous study [11], colistin resistance was identified only in 1.3% of isolates recovered from food-producing animals between 2005 and 2015. This study demonstrated that the colistin resistance rate was maintained below 2% for three consecutive years after 2015. Indeed, the prevalence of colistin-resistant isolates in this study was consistent with previous reports in Poland [12] but lower than other reports from Japan (48%) [13], China (42%) [14], and Cambodia (20%) [1].

Among a total of 185 colistin-resistant *E. coli* isolates determined in this study (47 from cattle, 90 from pigs, and 48 from chicken), PCR amplification detected *mcr*-*3* genes in 17 isolates: 2 from healthy pigs, 1 from a pig carcass, and 14 from diseased pigs (Table 1). *mcr-1* was the major colistin resistance determinant in *E. Couple* isolated from livestock, especially chickens in Korea since 2013 [11]. However, this study exhibited the emergence of *mcr-3* in livestock, especially in pigs since 2011. Notably, the majority of the *mcr*-*3* carrying isolates from diseased pigs were found between 2014 and 2018, highlighting a recent rise in prevalence compared to previous years. Recent studies reported a 38% [1] and 13% [15] prevalence of *mcr*-*3* among colistin-resistant pig isolates in Cambodia and Brazil, respectively. In addition, Fukuda et al. [13] identified the *mcr-3* gene in 8% of *E. coli* isolated from diseased pigs in Japan. *mcr-3-*carrying plasmids can stably persist by lowering fitness cost [16], suggesting careful monitoring of the *mcr-3* gene in Korean livestock.

The *mcr*-*3* genes were characterized as *mcr-3.1* in 15 isolates and *mcr*-*3.5* in 2 isolates (Table 1). Both of the *mcr*-3.5 isolates were identified in 2018. To the best of our knowledge, this is the first report of *mcr*-*3.5*-carrying *E. coli* in Korea, while these genes have been identified in *E. coli* from pigs and other sources in Europe and other Asian countries [17,18,19]. The *mcr-3* gene was transferred to *E. coli* J53 recipient strain from 53% of the *mcr*-*3*-carrying isolates as indicated by filter mating assay (Table 2), which is lower than Belaynehe et al. [2]. Agreeing with Zurfluh et al. [19], all *mcr*-*3*-carrying isolates were multi-drug resistant (MDR). Notably, the two *mcr*-*3.5*-carrying isolates from diseased pigs were resistant to ceftiofur. In addition, MDR in five *mcr*-*3-*carrying isolates was transferred to a recipient *E. coli*. Although we did not investigate other antimicrobial resistance genes, the co-existence of multiple resistant genes in the same or different plasmids could confer resistance to a broad range of antimicrobials [20].

PCR analysis presented five replicon types (IncP1, HI2, I1-α, IncM, and IncN). The *mcr*-*3.1* gene was identified predominantly in the IncHI2 plasmid, which is associated with the spread of MDR, including β-lactams and quinolones [14,21]. Agreeing with Li et al. [16], the *mcr*-*3.5* genes belonged to IncP plasmid. The less frequent plasmid replicon types in our study, such as IncI1-α, IncM, IncN, and IncP, were reported to co-harbor genes resistant to aminoglycosides, β-lactams, quinolones, and tetracyclines in *Enterobacteriaceae* [17,21,22].

Multi-locus sequence typing analysis revealed eight previously reported ST types: four ST1s, four ST10s, two ST42s, two ST641s, and each of ST29, ST101, ST3523, and ST4532 (Table 2). *E. coli* ST1, ST42, and ST641 isolates from diseased pigs of three farms showed similar patterns in the pulse-field gel electrophoresis results (Figure S1). Besides, ST1 and ST10 isolates carrying *mcr-3.1* gene were identified from farms located in different provinces. ST1, ST10, ST101, and ST641 *E. coli* isolates have already been identified in food-producing animals in several countries [11,12,15], suggesting its widespread distribution. Thus, a combination of clonal expansion and dissemination of plasmids carrying *mcr*-*3* variants contributed to the rise in the prevalence of *mcr*-*3* carrying *E. coli*.

We identified a total of 13 different virulence factor genes, with up to 5 of those in a single isolate (Table 2). The predominant virulence factors include the fimbrial adhesins (F18), the heat-labile (LT) or heat-stable (STb) enterotoxins, and virulence factors involved in diffuse adherence of *E. coli* (AIDA). *Mcr-3* positive strains isolated from diseased pigs were associated with enterotoxigenic *E. coli* (47.1%) and Shiga toxin-producing *E. coli* (23.5%), both expressing fimbrial adhesion (F18). These toxins and fimbrial genes are associated with porcine diarrhea and edematous disease [23,24].

**Figure 1:**
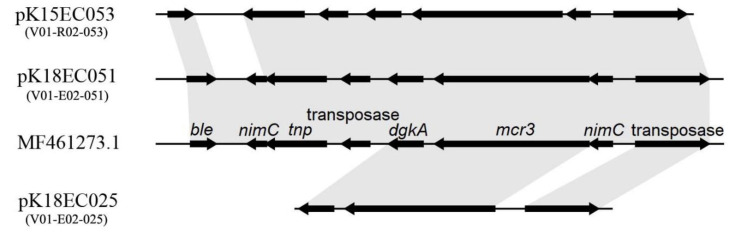
Comparison of the genetic environment of the *mcr*-*3* gene in plasmids pK18EC025, pK18EC051, and pK15EC053 with pWZR10 (MF461273.1). Arrows indicate the positions and directions of the genes. Regions with >99% homology are indicated by grey shading.

Whole-genome sequencing demonstrated that plasmids pK18EC051 (GenBank accession no. CP049300, 270.3 kb) and pK15EC053 (CP049087, 96.2 kb) shared a similar *mcr*-*3.1*-carrying region with plasmid pZR10 from pigs in China, but a gene encoding for 5-nitroimidazole based antimicrobials (*nimC*) was excluded from downstream of *mcr*-*3.1* gene in pK15EC053 (Figure 1). In contrast, only diacylglycerol kinase (*dgkA*) and transposase encoding genes were identified in the immediate downstream and upstream of the *mcr*-*3.5* gene, respectively, in pK18EC025 (CP049944, 60.2kb). The *mcr*-*3.5* variant in pK18EC025 differed from the *mcr*-*3.1* gene variant found in this study (pK18EC051 and pK15EC053) as well as the original *mcr*-*3* variant from China (pWJ1) by three amino acid substitutions (M23V, A457E, and T488I). Although the MIC of colistin was not altered by these substitutions, Yang et al. [16] demonstrated that *mcr-3.5* has higher fitness than *mcr-3.1*.

In conclusion, the proportion of *mcr*-*3*-carrying isolates is increasing in diseased pigs, presumably due to the horizontal and clonal dissemination. Therefore, active surveillance of *mcr*-carrying isolates is vital for preventing the spread of colistin resistance. In addition, a guideline that ensures prudent use of antimicrobials in pigs is urgently needed.

## Figures and Tables

**Table 1:** Prevalence of *mcr*-*3* in colistin-resistant *Escherichia coli* obtained from food-producing animals and animal carcasses from Korea.

Year (No. of Isolates)	Prevalence (%) of *mcr-3* Gene (no. of *mcr-3* Positive Isolates/ no. of Colistin-Resistant Isolates)												
	Cattle (*n* = 47)				Pigs (*n* = 90)				Chicken (*n* = 48)				Total (*n* = 185)
	Healthy	Carcasses	Diseased	Subtotal	Healthy	Carcasses	Diseased	Subtotal	Healthy	Carcasses	Diseased	Subtotal	
2005 (*n* = 693)	0 (0/1)	0 (0/0)	0 (0/0)	0 (0/1)	0 (0/0)	0 (0/0)	0 (0/0)	0 (0/0)	0 (0/0)	0 (0/0)	0 (0/0)	0 (0/0)	0 (0/1)
2006 (*n* = 744)	0 (0/0)	0 (0/0)	0 (0/0)	0 (0/0)	0 (0/5)	0 (0/0)	0 (0/0)	0 (0/5)	0 (0/0)	0 (0/0)	0 (0/0)	0 (0/0)	0 (0/5)
2007 (*n* = 744)	0 (0/12)	0 (0/0)	0 (0/0)	0 (0/12)	0 (0/17)	0 (0/0)	0 (0/0)	0 (0/17)	0 (0/0)	0 (0/0)	0 (0/0)	0 (0/0)	0 (0/29)
2008 (*n* = 559)	0 (0/16)	0 (0/0)	0 (0/0)	0 (0/16)	0 (0/6)	0 (0/0)	0 (0/0)	0 (0/6)	0 (0/1)	0 (0/0)	0 (0/0)	0 (0/1)	0 (0/23)
2009 (*n* = 641)	0 (0/5)	0 (0/0)	0 (0/0)	0 (0/5)	0 (0/7)	0 (0/0)	0 (0/0)	0 (0/7)	0(0/2)	0 (0/0)	0 (0/0)	0(0/2)	0 (0/14)
2010 (*n* = 1101)	0 (0/0)	0 (0/0)	0 (0/0)	0 (0/0)	0 (0/2)	0 (0/1)	0 (0/1)	0 (0/4)	0 (0/8)	0 (0/0)	0 (0/2)	0 (0/10)	0 (0/14)
2011 (*n* = 1276)	0 (0/4)	0 (0/2)	0 (0/0)	0 (0/6)	0 (0/2)	0 (0/1)	50 (1/2)	20 (1/5)	0 (0/3)	0 (0/4)	0 (0/5)	0 (0/12)	4.3 (1/23)
2012 (*n* = 1242)	0 (0/0)	0 (0/0)	0 (0/0)	0 (0/0))	50 (1/2)	0 (0/1)	0 (0/0)	33.3 (1/3)	0 (0/0)	0 (0/0)	0 (0/0)	0 (0/0)	33.3 (1/3)
2013 (*n* = 1078)	0 (0/2)	0 (0/0)	0 (0/0)	0 (0/2)	0 (0/0)	50 (1/2)	0 (0/3)	20 (1/5)	0 (0/2)	0 (0/1)	0 (0/0)	0 (0/3)	10 (1/10)
2014 (*n* = 1329)	0 (0/1)	0 (0/0)	0 (0/1)	0 (0/2)	0 (0/1)	0 (0/0)	50 (3/6)	42.9 (3/7)	0 (0/3)	0 (0/5)	0 (0/4)	0 (0/12)	14.3 (3/21)
2015 (*n* = 1169)	0 (0/0)	0 (0/0)	0 (0/1)	0 (0/1)	0 (0/0)	0 (0/0)	42.9 (3/7)	42.9 (3/7)	0 (0/2)	0 (0/1)	0 (0/0)	0 (0/3)	27.3 (3/11)
2016 (*n* = 1794)	0 (0/2)	0 (0/0)	0 (0/0)	0 (0/2)	0 (0/2)	0 (0/2)	0 (0/0)	0 (0/4)	0 (0/1)	0 (0/0)	0 (0/0)	0 (0/1)	0 (0/7)
2017 (*n* = 1218)	0 (0/0)	0 (0/0)	0 (0/0)	0 (0/0)	0 (0/5)	0 (0/1)	0 (0/0)	0 (0/6)	0 (0/0)	0 (0/0)	0 (0/0)	0 (0/0)	0 (0/6)
2018 (*n* = 1043)	0 (0/0)	0 (0/0)	0 (0/0)	0 (0/0)	50 (1/2)	0 (0/0)	58.3 (7/12)	57.1(8/14)	0 (0/0)	0 (0/1)	0 (0/3)	0 (0/4)	44.4 (8/18)
Total	0 (0/43)	0 (0/2)	0 (0/2)	0 (0/47)	3.9 (2/51)	12.5 (1/8)	45.2 (14/31)	18.9 (17/90)	0 (0/22)	0 (0/12)	0(0/14)	0(0/48)	9.2 (17/185)

**Table 2:** Characteristic of the *mcr-3* positive *Escherichia coli* from healthy and diseased pigs, and pig carcasses in Korea.

Isolates	Source	Farm ID	Year	Province	MIC of Colistin (µg/mL)	MCR-3 Variant Type	Resistance Pattern ^a,b^	Transfer-Ability	Replicon Type of Transconjugant Plasmid	Multiloccus Sequence Type	PULSOTYPE	Virulence Factors
V08-R02-015	diseased	GB-1	2011	Gyeongbuk	16	*mcr*-3.1	AMP CHL CIP GEN NAL STR FIS TET SXT	/		3523	A	F18/LT/STb/EAST
V04-A02-010	healthy	CN-1	2012	Chungnam	8	*mcr*-3.1	AMP CHL GEN STR FIS TET SXT	+	HI2	4532	B	
V05-S02-016	carcass	CN-2	2013	Chungnam	8	*mcr*-3.1	CHL NAL STR FIS TET	/		101	C	EAST
14D084	diseased	GB-2	2014	Gyeongbuk	16	*mcr*-3.1	AMPCHLGEN NAL FIS TET SXT	+	HI2, I1-α	1	D	F18/Stx2e /AIDA
14D084-2	diseased	GB-2	2014	Gyeongbuk	16	*mcr*-3.1	AMP GEN NAL FIS TET SXT	/		1	D	F18/Stx2e /AIDA
14D085	diseased	GB-3	2014	Gyeongbuk	16	*mcr*-3.1	AMP CHL GEN NAL FIS TET SXT	/		1	D	F18/Stx2e /AIDA
V01-R02-019	diseased	CB-4	2015	Chungbuk	16	*mcr*-3.1	AMP CHL STR FIS TET SXT	+	HI2	ND ^c^	E	
V01-R02-020	diseased	CB-4	2015	Chungbuk	8	*mcr*-3.1	AMP CIP CHL NAL STR FIS TET SXT	/		10	- ^d^	
V01-R02-053	diseased	GG-1	2015	Gyeonggi	16	*mcr*-3.1	AMP CHL FIS TET SXT	+	M	1	D-1	F18/Stx2e/AIDA
V01-A02-017	healthy	GN-3	2018	Gyeongnam	16	*mcr*-3.1	AMP CHL CIP NAL STR FIS TET SXT	/		10	L	LT/ STb/EAST
V01-E02-088	diseased	GN-4	2018	Gyeongnam	16	*mcr*-3.1	AMP CHL CIP GEN NAL STR FIS TET SXT	/		10	L	LT/ STb/EAST
V01-E02-090	diseased	GN-4	2018	Gyeongnam	>16	*mcr*-3.1	AMPCHL CIP GEN NAL STR FIS TET SXT	+	HI2, I1-α, N	29	M	eae/paa
V01-E02-023	diseased	GB-4	2018	Gyeongbuk	4	*mcr*-3.5	AMP CHL XNL STR FIS TET SXT	+	P1, I1-α	42	N	F18/LT/STb/EAST
V01-E02-025	diseased	GB-4	2018	Gyeongbuk	8	*mcr*-3.5	AMP CHL XNL STR FIS TET SXT	+	P1	42	N	F18/LT/STb/EAST
V01-E02-049	diseased	GN-5	2018	Gyeongnam	>16	*mcr*-3.1	AMC AMP FOX CHL CIP GEN NAL STR FIS TET SXT	/		10	L	F18/LT/STb/EAST/AIDA
V01-E02-050	diseased	GN-5	2018	Gyeongnam	8	*mcr*-3.1	AMP CHL GEN STR FIS TET SXT	+	HI2	641	O	STb/EAST/AIDA
V01-E02-051	diseased	GN-5	2018	Gyeongnam	8	*mcr*-3.1	AMP CHL GEN STR FIS TET SXT	+	HI2	641	O	STb/EAST/AIDA

^a^ AMC, amoxicillin/clavulanic acid; AMP, ampicillin; FOX, cefoxitin, XNL, ceftiofur; CHL, chloramphenicol; CIP, ciprofloxacin; GEN, gentamicin; NAL, nalidixic acid; STR, streptomycin; FIS, sulfisoxazole; TET, tetracycline; SXT, trimethoprim/sulfamethoxazole. ^b^ The underlined resistance markers were transferred to the recipient *E. coli* J53 strain by conjugation. ^c^ Not determined. ^d^ *Xbal* macrorestriction analysis yielded no DNA banding patterns in V01-R02-020 *E. coli* strain due to constant autodigestion of the genomic DNA during agarose plug preparation, and thus, a cluster formed by this strain is excluded. +: *mcr*-3 gene was transferred to E. coli J53 recipient strain. /: *mcr-3* gene was not transferred to E. coli J53 recipient strain.

## Supplementary Materials

The following are available online at https://www.mdpi.com/2076-2607/8/10/1538/s1. **Figure S1**: X*bal*-digested pulsed-field gel electrophoresis patterns of *mcr*-*3* carrying *E. coli* strains isolated from healthy pigs, pig carcasses, and diseased pigs in Korea. X*bal* macrorestriction analysis yielded no DNA banding patterns in V01-R02-020 *E. coli* strain due to constant autodigestion of the genomic DNA during agarose plug preparation, and thus, a cluster formed by this strain is excluded. (ND, not determined).
