# Supplementary figures and images for: Emergence of mcr-3 carrying Escherichia coli in Diseased Pigs in South Korea
Source: Microorganisms. 2020 Oct 6;8(10):1538. doi: 10.3390/microorganisms8101538 (PMC7650651; doi:10.3390/microorganisms8101538)

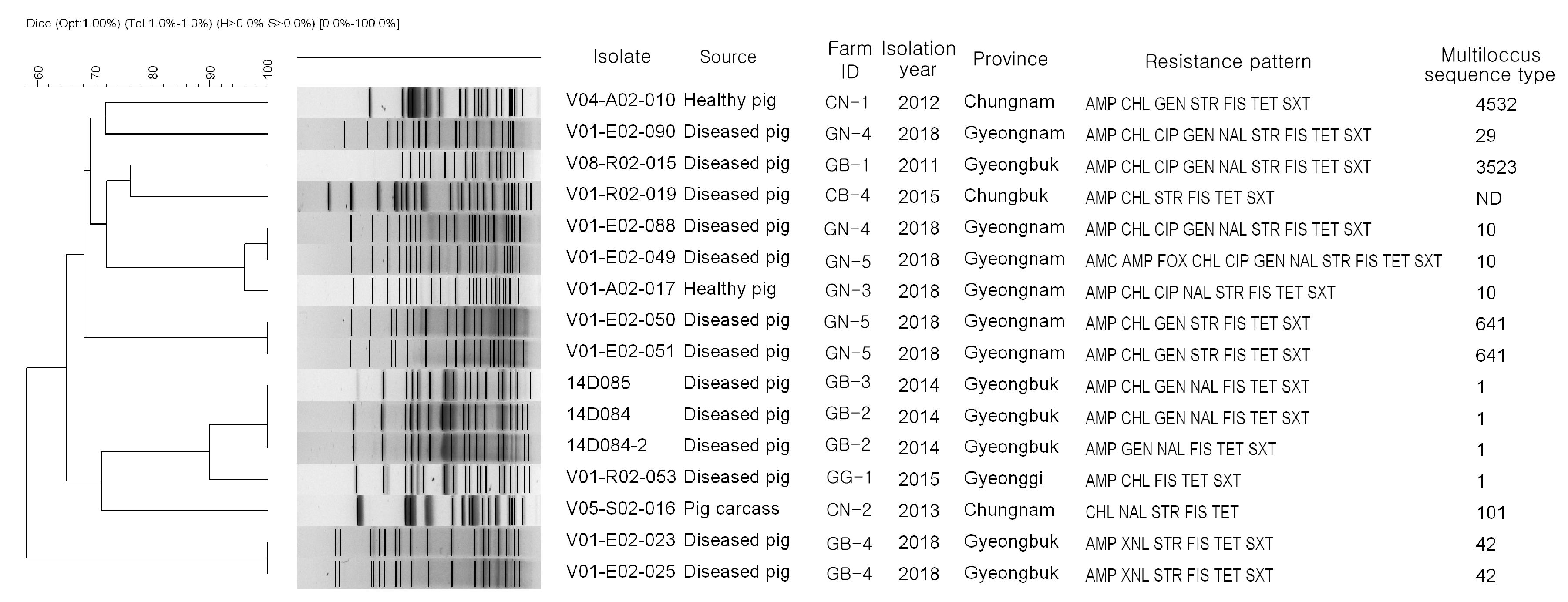

Supplement: Supplementary file 1 [file microorganisms-08-01538-s001.png]
